# Highly Sensitive Detection of Carcinoembryonic Antigen *via* an Electrochemical Platform Fabricated by AuNPs/Streptavidin/Reduced Graphene Oxide

**DOI:** 10.3389/fchem.2022.898924

**Published:** 2022-05-12

**Authors:** Yang Guo, Liang Feng

**Affiliations:** Department of Breast Surgery, The First Affiliated Hospital of China Medical University, Shenyang, China

**Keywords:** electrochemical immunosensor, reduced graphene oxide, AuNPs, CEA, signal amplification strategy

## Abstract

Tumor markers are one of the important indicators for early cancer diagnosis. As a new analytical method, electrochemical immunosensing analysis has the advantages of high sensitivity, good selectivity, and rapid detection, which is of great significance for the detection of tumor markers. In this work, an AuNP/reduced graphene oxide (AuNP/rGO) composite was synthesized. We used it for electrochemical sensor fabrication with the assistance of the biotin–streptavidin protein (SA) system to further amplify the signal to achieve sensitive detection of carcinoembryonic antigen (CEA). In addition, AuNPs have been incorporated due to their good electrical conductivity and biocompatibility, which can accelerate electron transfer at the electrode interface and improve the loading capacity to capture antibodies. The fabricated AuNPs/SA/rGO has a large working surface area and high material utilization ratio, which improves the catalytic capacity of H_2_O_2_ reduction and effectively amplifies the current signal. The linear range of the response current signal of the sensor toward the CEA concentration is 20 fg/ml to 200 ng/ml, and the limit of detection can achieve 6.2 fg/ml. In addition, the fabricated immunosensor has good reproducibility, selectivity, and stability.

## Introduction

Among the problems about human health, cancer has the characteristics of being painful and difficult to cure, has long treatment cycle, and is expensive, which seriously threaten people’s life, health, and safety, as well as the whole family life. Early cancer is generally not easy to detect, but early cancer detection is the golden period of treatment. The cure rate of early cancer can reach more than 80% (Mohammadi et al., 2019; Wang H et al., 2020). Clinical diagnosis of cancer mainly uses imaging detection methods, such as B ultrasound, CT, magnetic resonance imaging (MRI), and other methods, but these require expensive equipment and complex operation. When a site or tissue becomes cancerous, corresponding tumor markers will be generated, and these tumor markers will generally enter the blood and body fluids. Therefore, the detection of tumor markers in blood samples is one of the simple and effective methods for early diagnosis of cancer such as breast cancer (Filik and Avan, 2019; Vajhadin et al., 2020; Zhang et al., 2021). Tumor markers are molecules that are characterized in malignant tumor cells with main components including proteins and sugars, which are released by their own synthesis or generated by the body’s reaction. In recent years, great progress has been made in the study of tumor markers, and many tumor markers have been discovered and applied in the detection of malignant tumors (Wang J et al., 2020; Karimi-Maleh et al., 2021b; Wei et al., 2021). The abnormal increase of tumor markers can indicate the occurrence of tumor, and the sensitive detection of tumor markers is of great significance for the diagnosis, treatment, and prognosis monitoring of cancer.

Carcinoembryonic antigen (CEA) is a broad-spectrum tumor marker found in fetal and colon tissues (Sadighbayan et al., 2019; Wen et al., 2020). The normal value of serum CEA in normal people is less than 5 μg/L, but breast cancer will lead to an increase in the serum CEA concentration. CEA plays an important role in diagnosis and treatment, prognosis monitoring, and recurrence assessment of cancer (El Aamri et al., 2020; Chen et al., 2021). Sensitive detection of tumor markers has always been one of the goals of researchers. In recent years, the detection methods of tumor markers have been optimized, such as radioimmunoassay, enzyme-linked immunosorbent assay, and electrochemical immunosensor (Karaman, 2021; Karimi-Maleh et al., 2022a; Karimi-Maleh et al., 2022c; Mehrizi et al., 2022; Mubashir et al., 2022). The electrochemical immunosensor performs qualitative or quantitative analysis by measuring the electrochemical or electrical properties of the analyte (Sfragano et al., 2021). Compared with traditional analytical techniques, the electrochemical immunosensor has the advantages of low cost, fast analytical speed, and simple operation (Song et al., 2019; Mani et al., 2021).

Nanomaterials are introduced into the field of electrochemical analysis. On the one hand, nanomaterials are applied to the surface modification of the electrode, which can greatly improve the conductivity of the electrode (Cui et al., 2019; Sharifi et al., 2019). At the same time, it can catalyze the accelerated reaction process, shorten the time of analysis and detection, and immobilize more antibodies or antigens to achieve rapid and sensitive detection of the analyte. Graphene is a typical two-dimensional nanomaterial consisting of a single layer of carbon atoms (Xu et al., 2021). The special two-dimensional structure of graphene makes it have a great theoretical specific surface area, ultra-high electron mobility, excellent optical and mechanical properties, and other characteristics (Shahzad et al., 2020). This makes graphene have broad application prospects in sensors, supercapacitors, luminescent electronic components, batteries, and other fields. In order to further expand the application of graphene in electrochemical sensors, a lot of work has been carried out to further optimize the performance of graphene by means of doping other materials, chemical functionalization, and synthesis of composite materials (Reddy et al., 2020). Gold is the most chemically stable metal, with good electrical conductivity and biocompatibility, and can be combined with biologically active proteins, enzymes, and other molecules without destroying their activity (Karimi-Maleh et al., 2021a; Wang et al., 2021; Zheng et al., 2021; Karimi-Maleh et al., 2022b; Pothipor et al., 2022). Gold nanomaterials are often modified on the electrode surface as substrate materials to immobilize antigens or antibodies and provide a good channel environment for electron transfer on the electrode surface. In addition, gold nanomaterials have the characteristics of easy functionalization (Akbari jonous et al., 2019; Tran et al., 2021; Fu et al., 2022). Therefore, gold nanomaterials can also be combined with other materials to achieve the effect of amplifying electrical signals. In this study, graphene oxide (GO) and AuNPs were used as electroactive materials for the signal amplification of the sensor. In order to improve the sensitivity of the immunosensor, a biotin–streptavidin (SA) system was adopted (Xia et al., 2020; Liu et al., 2021a; Liu et al., 2021b; Xia et al., 2021). The biotin-SA CEA antibody (Bio-Ab) was captured because of the high affinity between biotin and SA. After the non-specific sites were sealed with bovine serum albumin (BSA), the CEA to be measured was bound to the immunosensor through antigen- and antibody-specific reactions. Finally, the constructed electrochemical immunosensor obtained an excellent electrochemical response signal by reducing H_2_O_2_. The fabricated electrochemical immunosensor has the advantages of high sensitivity, high specificity, good repeatability, and wide detection range.

## Materials and Experiments

### Materials and Instrument

CEA and CEA antibodies were purchased from Shanghai Lingchao Biotechnology Co., Ltd. Bovine serum albumin (BSA, 96–99%) was purchased from Sigma Reagents Ltd. Chloroauric acid (HAuCl_4`_4H_2_O) was purchased from Beijing Sigma-Aldrich China Co., Ltd. Streptavidin protein (SA) and biotin-labeled cancer embryo antigen monoclonal antibody (anti-CEA/Biotin) were purchased from Beijing Boaosen Biotechnology Co., Ltd. Sodium borohydride (NaBH_4_) was purchased from Shanghai Energy Chemical Co., Ltd. Reduced graphene oxide (rGO) was purchased from Jiangsu Xianfeng Nanomaterials Technology Co., Ltd. H_2_O_2_ was purchased from Chongqing Chuandong Chemical Co., Ltd. The pH 6.8 phosphate buffer solution (PBS, composed of 0.1 M Na_2_HPO_4_, 0.1 M KH_2_PO_4_, and 0.1 M KCl) was used as the working buffer solution. Then, 0.01M PBS (consisting of 8 mM Na_2_HPO_4_, 2 mM KH_2_PO_4_, 0.1 M NaCl, and 3 mM KCl) was used as the diluent of CEA, SA, and anti-CEA/biotin. All other reagents are analytically pure and can be used directly without purification.

### Preparation of AuNPs and AuNPs/rGO

First, 1.0 ml of HAuCl_4_ 4H_2_O with a concentration of 1.0 wt% was added to 99.0 ml water and stirred in a magnetic stirrer under 100°C oil bath to boil. Then, 2.5 ml of sodium citrate solution with a concentration of 1.0 wt% was added, and magnetic stirring was continued for 15 min to obtain a wine red AuNP solution.

AuNPs/rGO was prepared by using a one-step solvothermal method. A measure of 10.0 ml of AuNP solution with a concentration of 10.0 mg/ml was added to 50.0 ml of GO solution with a concentration of 1.0 mg/ml. After 10 min of magnetic stirring, the resulting mixture was transferred to a teflon-lined high-pressure reactor, and a solvothermal reaction was carried out at 130°C for 3 h. The AuNPs/rGO was obtained by freeze-drying the black precipitate obtained by continuous washing and centrifugation with water and ethanol five times.

### Fabrication of the Electrochemical Immunosensor

**FIGURE 1:**
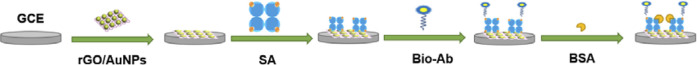
Scheme of the preparation of BSA/Bio-Ab/SA/AuNPs/rGO/GCE.

First, the glassy carbon electrode (GCE) was polished successively with 0.3 and 0.05 μm alumina powder. Then, the GCE was ultrasonically washed with water and then ethanol for 5 min each. After GCE drying at room temperature, 8 μl of 2 mg/ml AuNPs/rGO was added to the surface of the electrode and dried in air. Then, 10 μl of SA with a concentration of 1 μg/ml was dropped onto the surface of the modified electrode, and SA bonded to the modified electrode through the Au-NH_2_ bond. The electrodes were stored and incubated at 4°C for 12 h to make the SA protein bind to AuNPs/rGO more effectively. Next, 10 μl of biotin-modified carcinoembryonic antigen antibody (Bio-Ab) was dropped onto the surface of the prepared electrode and incubated at 37°C for 60 min. This step allows the Bio-Ab to be immobilized to the electrode by the high affinity between SA and biotin. Then, 6 μL of BSA solution (1% w/v) was added and incubated at 37°C for 40 min to block the non-specific binding. After each modification step, the electrode was washed with PBS solution to remove unbound SA, Bio-Ab, and BSA. Figure 1 shows the scheme of the sensor preparation.

### CEA Detection

A volume of 10 μl of CEA standard solution with different concentrations (ranging from 0.1 pg/ml to 1 μg/ml) was added to the modified electrode and incubated at 37°C for 60 min. The uncombined CEA was removed by washing with PBS. All electrochemical tests were performed on an electrochemical workstation in a conventional three-electrode system. Cyclic voltammetry (CV) and electrochemical impedance (EIS) measurements were carried out in 5 mM [Fe(CN)_6_]^3−/4−^ solution containing 0.1 M KCl. The electrochemical impedance method was performed at the amplitude of the frequency range 0.1–10^5^ Hz and 10 mV. The timing current method (i-t method) involves immersing the modified working electrode in a phosphate buffer solution used as a working buffer solution. At room temperature and voltage −0.4 V, after the background current was stable, 10 μl of 2 M H_2_O_2_ was added into a small beaker containing 10 ml of working buffer solution (pH 6.8), and the difference in the change of the electrochemical signal in the i-t curve was recorded.

## Results and Discussion

**FIGURE 2:**
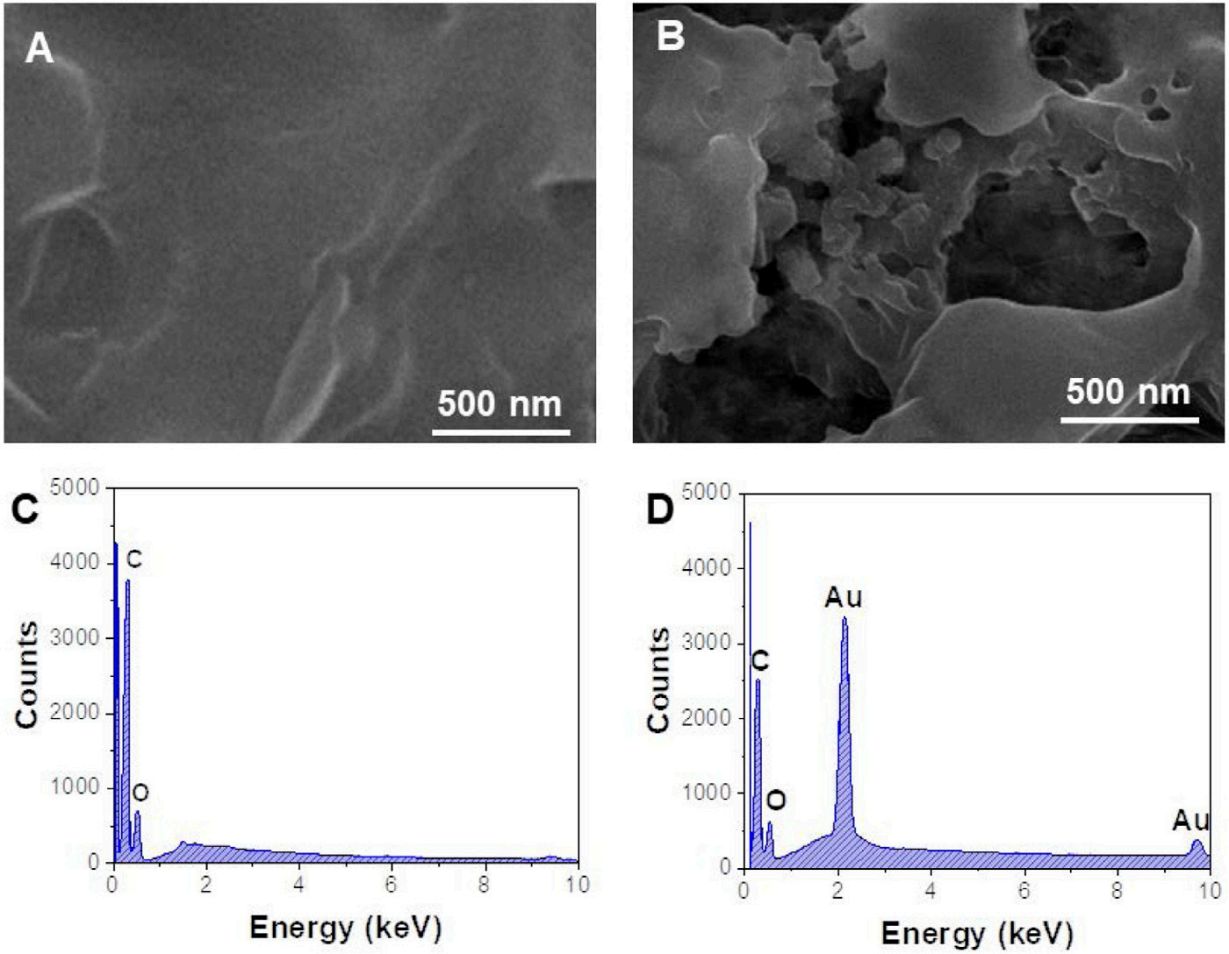
SEM image of **(A)** rGO and **(B)** AuNPs/rGO. EDX spectra of **(C)** rGO and **(D)** AuNPs/rGO.

The morphology of the prepared nanomaterials was characterized by SEM. As shown in Figure 2A, rGO has a continuous smooth surface structure accompanied by a folded paper shape, with a large specific surface area, and is a good substrate for loading a large number of nanoparticles. After rGO was combined with AuNPs, the SEM image (Figure 2B) showed that a large number of spherical metal nanoparticles were obviously attached to the lamellar structure of rGO (Amir et al., 2020). Figures 2C,D are EDS spectra of rGO and AuNPs/rGO. Compared with Figure 2C, the characteristic element peaks corresponding to Au can be clearly observed in Figure 2D, which means AuNPs are successfully anchored on rGO.

**FIGURE 3:**
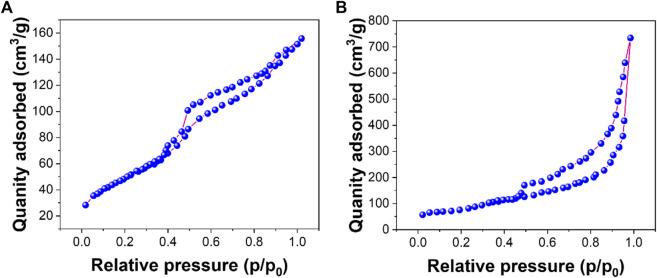
N_2_ adsorption–desorption isotherm: **(A)** rGO; **(B)** AuNPs/rGO.

Figures 3A,B show nitrogen adsorption–desorption isotherms for rGO and AuNPs/rGO, respectively. According to the nitrogen adsorption–desorption experiment, the average pore size of rGO is 5.22 nm with a BET surface area of 172.61 m^2^/g. The average pore size of AuNPs/rGO is 12.51 nm with a BET surface area of 366.12 m^2^/g. The average pore size and BET surface area of AuNPs/rGO are significantly larger than those of rGO, which can be attributed to the large pore structure and surface area of AuNPs inserted into the rGO lamellar structure as spacers (Liu S et al., 2021). This effectively proves that AuNPs/rGO is successfully prepared, and the prepared composite has an obvious spacer structure.

**FIGURE 4:**
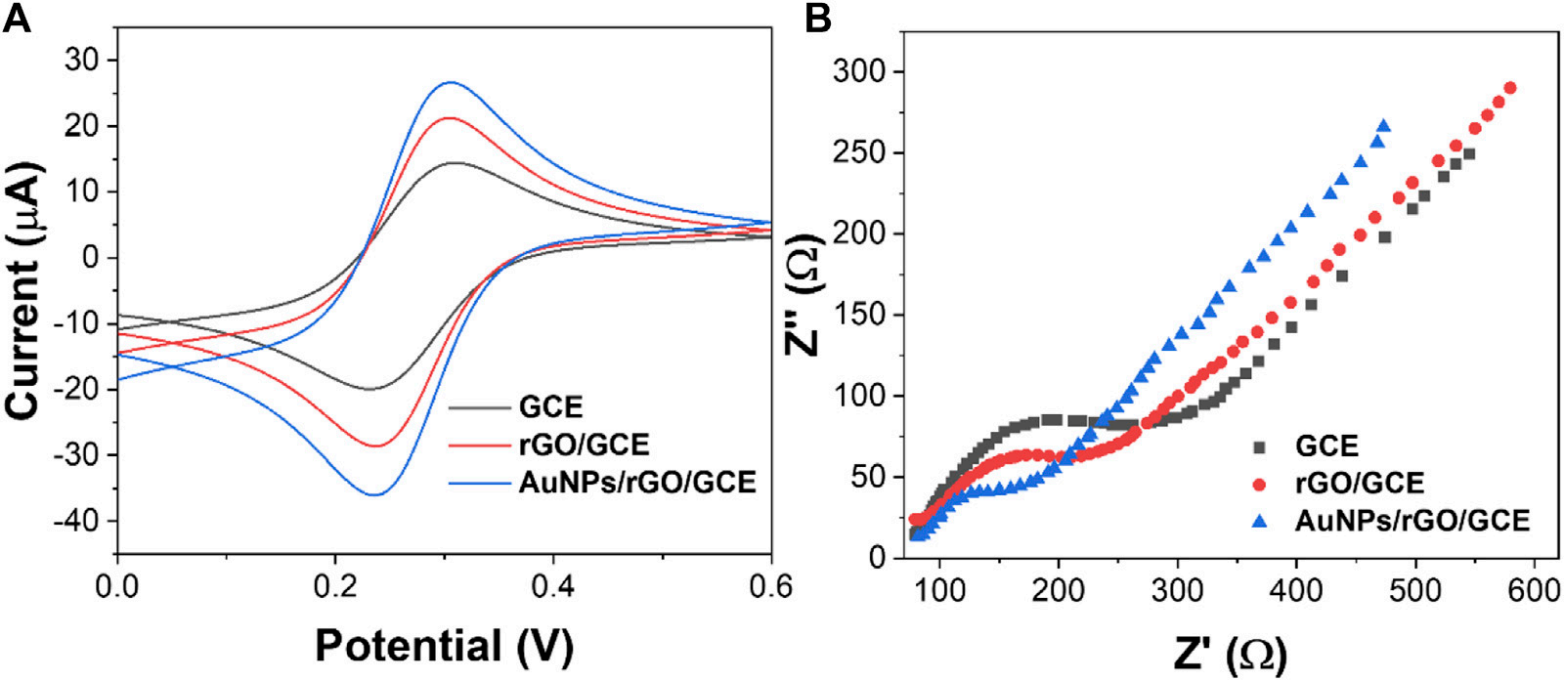
**(A)** CV and **(B)** EIS spectra of GCE, rGO/GCE, and AuNPs/rGO/GCE.

Electrochemical characterization of rGO and AuNPs/rGO was conducted by CV and EIS. As shown in Figure 4A, the redox peak current of rGO is larger than that of bare GCE. When using an AuNP/rGO-modified electrode, the redox peak current is the most obvious compared with rGO and bare GCE. CV test results show that AuNPs/rGO has better electrocatalytic activity than rGO. This can be attributed to the spacer structure formed by AuNPs inserted into the rGO lamella, exposing more catalytic active sites (Dehghani et al., 2019).

In addition, the conductivity of AuNPs/rGO and rGO was tested by EIS. The semicircle of the high-frequency region in the EIS corresponds to surface transfer resistance (R_et_). As can be seen from Figure 4B, when the electrode surface is modified with rGO, the semicircle diameter at a high frequency is smaller than that of bare GCE. This shows that rGO can effectively accelerate electron transfer (Nazarpour et al., 2020). When the electrode surface was modified with AuNPs/rGO, the semicircle diameter at high frequency was smaller, and the R_et_ was smaller than that of rGO. After AuNPs were inserted into rGO as a spacer, the electron transfer rate of AuNPs/rGO is faster and the conductivity is better.

**FIGURE 5:**
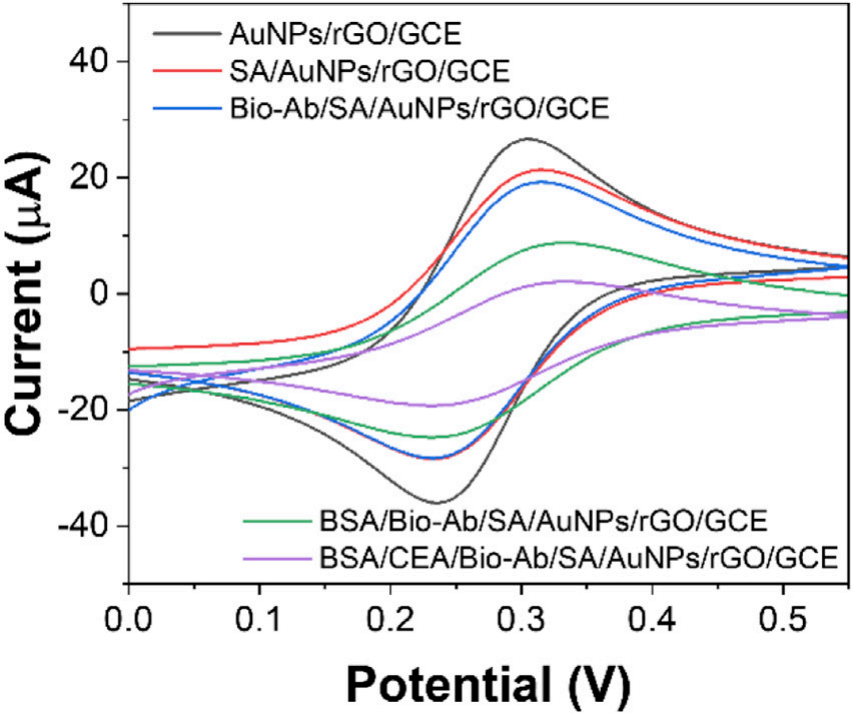
CV curves of AuNPs/rGO/GCE, SA/AuNPs/rGO/GCE, Bio-Ab/SA/AuNPs/rGO/GCE, BSA/Bio-Ab/SA/AuNPs/rGO/GCE, and CEA/BSA/Bio-Ab/SA/AuNPs/rGO/GCE.

The sensor assembly was also characterized by CV (Figure 5). After incubating SA on the AuNPs/rGO/GCE surface, the peak current decreased significantly because the non-electroactive protein SA blocked the electron transfer on the electrode. Then, Bio-Ab binds to SA, and the non-electroactive antibody–protein Bio-Ab further increases the resistance on the electrode surface and decreases the redox peak current (Avan and Filik, 2020). Similarly, when non-specific sites of Bio-Ab/SA/AuNPs/rGO/GCE are blocked with BSA, the peak current is again reduced. Then, under the antigen–antibody reaction, high electrical resistance is produced. When the antigenic protein CEA acts as a mass transfer barrier and binds inert electrons with Bio-Ab, the peak current decreases to a minimum. These results indicate that each step of the immunosensor manufacturing process is successfully fabricated (Ghanbari et al., 2019).

**FIGURE 6:**
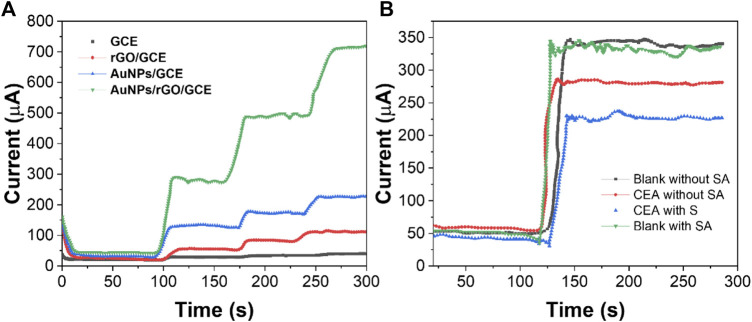
**(A)** i-t curves of GCE, AuNPs/GCE, rGO/GCE, and AuNPs/rGO/GCE. **(B)** i-t curves of the immunosensor modified with or without SA.

The sensitivity of the electrochemical immunosensor depends on the electrocatalytic activity of the modified material on the working electrode surface (Fan et al., 2020). In order to study the mechanism of signal amplification, we modified the GCE surface with different materials and tested it with the i-t curve of timing current. As shown in Figure 6A, AuNPs/GCE has a small but stable response current value, which indicates that AuNPs, as a substrate material, can adhere uniformly to the electrode surface and accelerate the electron transfer between the electrode surface and the electrolyte (Poo-arporn et al., 2019). When rGO is modified on the GCE surface, the response current value is very small. When AuNPs are loaded on rGO, the response current is larger than AuNPs/GCE. This can be attributed to the synergistic effect between AuNPs and rGO on the catalytic performance of the H_2_O_2_ reduction reaction that greatly improved (Hashemi et al., 2020). Therefore, AuNPs/rGO/GCE can greatly improve the sensitivity of the immunosensor.

In order to verify the signal amplification effect of the biotin-SA system in the immunosensor, we conducted a comparative experiment with or without the immunosensor added with SA. Due to the high affinity of the biotin-SA system and the characteristic that one SA molecule can combine with four biotin molecules (Luong and Vashist, 2019), more Bio-Ab can be combined when using AS to modify the immunosensor. When detecting CEA with the same content, the SA-modified immunosensor showed a higher difference in the electrochemical response than the immunosensor without SA modification (Figure 6B). The aforementioned results show that the signal amplification strategy can further improve the sensitivity of CEA detection.

**FIGURE 7:**
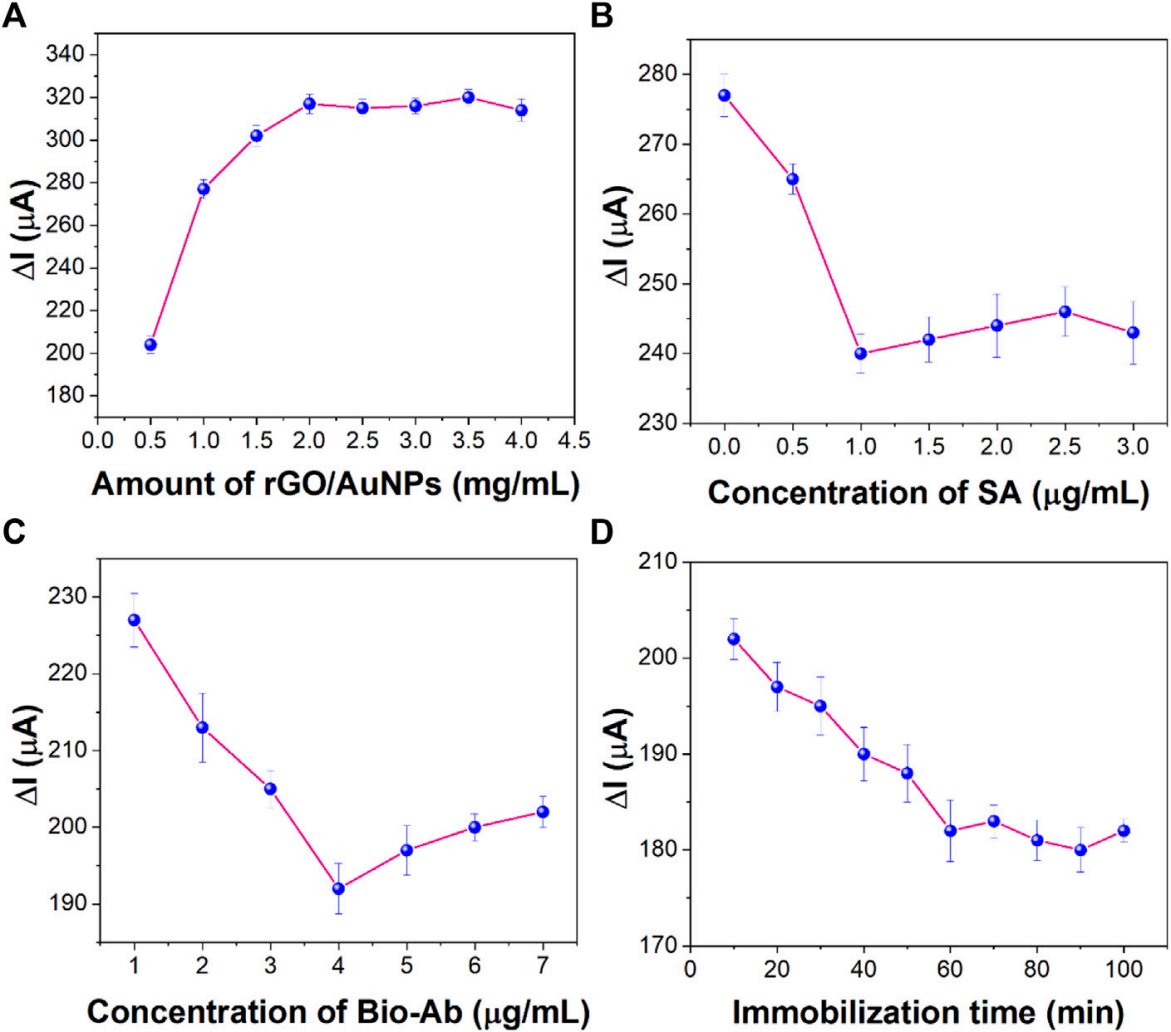
Effect of **(A)** concentration of rGO/AuNP nanocomposites, **(B)** concentration of SA, **(C)** concentration of Bio-Ab, and **(D)** immobilization time of CEA on the sensing performance (*n* = 3).

The concentration of AuNPs/rGO is an important factor affecting the surface area of the electrode and the conductivity of the sensor. As shown in Figure 7A, the electrochemical signal gradually increased (from 0.5 to 2.0 mg/ml) as the concentration of the incubated nanocomposites increased and then remained stable. Therefore, 2.0 mg/ml was selected as the optimal concentration of AuNPs/rGO nanocomposites.

SA concentration is a key factor that has important influence on the performance of the immunosensor and Bio-AB immobilization. As shown in Figure 7B, the electrochemical signal decreased with the increase in the SA concentration. The electrochemical signal tended to be stable at the concentration of 1 μg/ml, indicating that the amount of SA on the surface of the immunosensor reached the maximum. Therefore, 1 μg/ml was selected as the best concentration of SA.

In order to synthesize more Bio-Ab immobilized on the electrode, the concentration of Bio-Ab is also an important parameter affecting the performance of the sensor. Figure 7C shows that the concentration of Bio-Ab changes from 2 to 4 μg/ml, and the electrical signal decreases significantly. However, when the concentration is greater than 4 μg/ml, the signal remains stable. This reflected that the biotin-labeled antibody reached its maximum value by binding to SA. Therefore, 4 μg/ml was used as the optimal concentration of Bio-Ab.

The specific recognition and immobilization of the target CEA are key steps in the whole sensor construction process. As shown in Figure 7D, as the incubation time of antigen increases, the current decreases rapidly and then remains stable after 60 min. This indicates that maximum immobilization of CEA was achieved through antigen–antibody-specific recognition at this time. Therefore, 60 min was chosen as the best incubation time for CEA.

**FIGURE 8:**
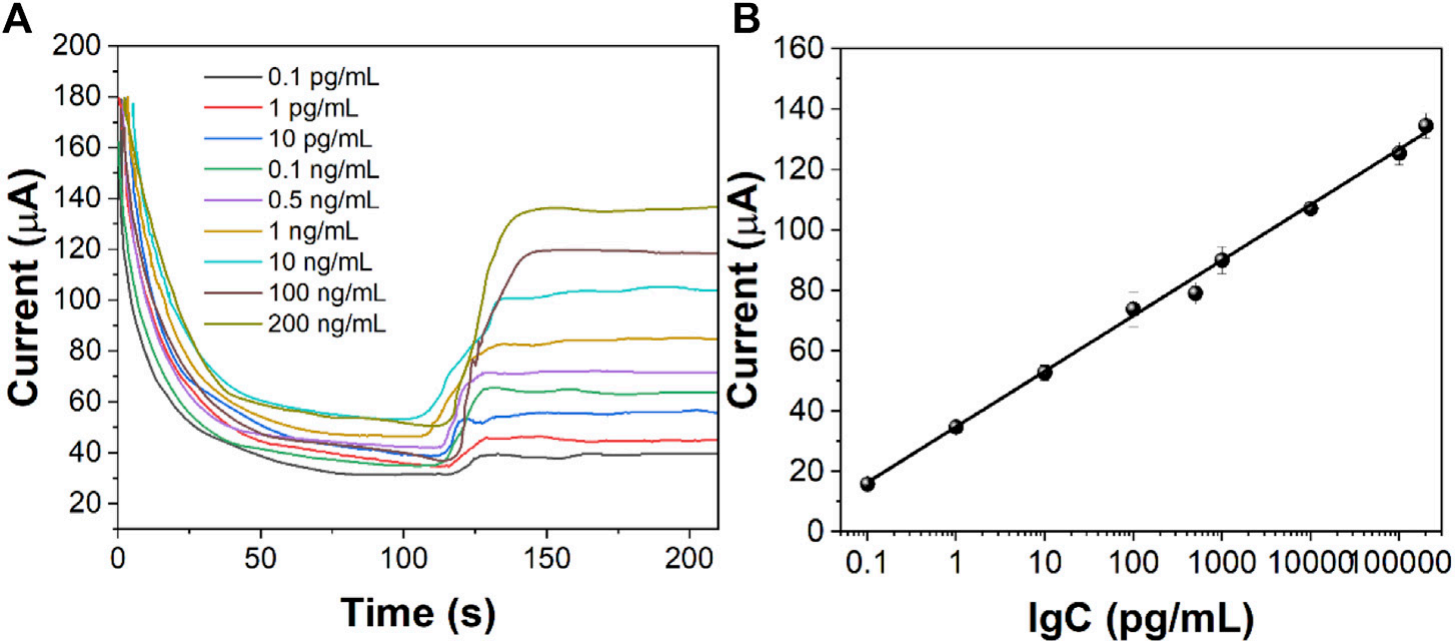
**(A)** i-t curves of the immunosensor to different concentrations of CEA. **(B)** Calibration curve of CEA concentration against the value of the immunosensor.

Different concentrations of CEA were detected by the i-t curve method under optimum experimental conditions. Figure 8A shows the response current results of different concentrations of CEA. The response current increases with the increase in the CEA concentration. There is a good linear relationship between the log value of current (∆I) and CEA concentration in the range of 20 fg/ml–200 ng/ml. The limit of detection limit (LOD) was calculated to be 6.2 fg/ml. The values of points in Figure 8B are obtained by the formula ∆I = I_0_−I_t_. I_0_ is the stable background current value, and I_t_ is the stable current value obtained after injecting H_2_O_2_. The linear regression equation obtained is ∆I = −15.419lgC −80.611 (*R* ^2^ = 0.9978). The results showed that the detection range of CEA was wide and the LOD was low. Table 1 shows the sensing performance of the proposed electrochemical sensor with other electroanalytical methods.

**TABLE 1:** Linear range and LOD obtained using a proposed immunosensor and other sensors reported for CEA sensing.

Sensor	Linear range	LOD	Reference
CEA/rGO/GCE	0.1–5 ng/ml	0.05 ng/ml	Jozghorbani et al. (2021)
CPS@PANI@Au	0.006–12 ng/ml	1.56 pg/ml	Song et al. (2021)
[Ag-Ag_2_O]/SiO_2_	0.5–160 ng/ml	0.14 ng/ml	Yuan et al. (2010)
CS–CNT–GNP nanocomposite film	0.1–2 ng/ml	0.04 ng/ml	Gao et al. (2011)
Ag@SiO_2_ NPs	0.5–10 ng/ml	0.01 ng/ml	Singh et al. (2021)
Cu-MOF-TB/PDA	0.02 pg/ml–200 ng/ml	3.0 fg/ml	Liu et al. (2020)
Pd-V_2_O_5_/CNT	0.5 pg/ml–25 ng/ml	170 fg/ml	Han et al. (2016)
CD-GN-Cu@Au	0.1 pg/ml–20 ng/ml	20 fg/ml	Gao et al. (2015)
Cu_2_O-Au	2 pg/ml–20 ng/ml	200 fg/ml	Qin et al. (2018)
CEA/BSA/Bio-Ab/SA/AuNPs/rGO/GCE	20 fg/ml–200 ng/ml	6.2 fg/ml	This work

**FIGURE 9:**
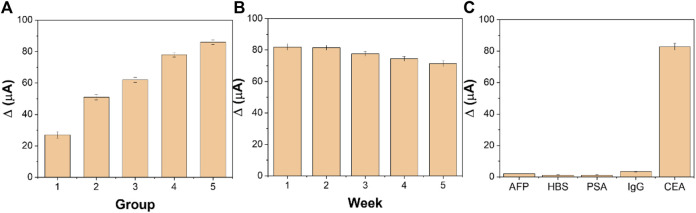
**(A)** Reproducibility, **(B)** stability, and **(C)** specificity of the fabricated immunosensor (*n* = 3).

Reproducibility is an important index to evaluate the accuracy of the designed electrochemical immunosensor. We prepared five groups of immunosensor, five in each group, to detect CEA at the concentrations of 1 pg/ml, 10 pg/ml, 100 pg/ml, 1 ng/ml, and 10 ng/ml, respectively. Under the optimum experimental conditions, the immunosensor was tested by the time-current i-t curve method. Figure 9A shows the average response current obtained by testing the five groups of electrodes, with relative standard deviations (RSD) of 3.11%, 2.27%, 1.98%, 2.06%, and 2.24%, respectively. The results show that the designed electrochemical immunosensor has good reproducibility.

The stability of the immunosensor is tested by periodically measuring the current response. Five immunosensors were prepared to detect 1 ng/ml CEA under optimal experimental conditions. As shown in Figure 9B, the designed immunosensor changed only 3.59% after 2 weeks and 6.61% after 3 weeks. After 5 weeks of storage, the current value decreased by 10.01% compared with the initial period. The decrease in the response current value may be due to the accumulation of gradual inactivation of CEA and CEA antibodies. It is proven that the designed immunosensor has good stability.

The interference experiments of AFP, HBS, PSA, and IgG were carried out to investigate the selectivity of the designed immunosensor. The CEA of 1 and 10 ng/ml interference solution was determined by the designed immunosensor. Compared with the detection results of pure CEA, the current change caused by interfering substances in Figure 9C is less than 5%, indicating that the designed electrochemical immunosensor has excellent selectivity and specificity.

## Conclusion

A highly sensitive CEA electrochemical immunosensor was successfully constructed based on the SA-biotin system and rGO/AuNP nanocomposites as both substrate modification materials and signal amplification molecules. rGO/AuNPs not only significantly improved the conductivity but also showed outstanding electrocatalytic activity in reducing hydrogen peroxide. In addition, the introduction of the biotin-SA system also contributes to the enhancement of the sensitivity of the immunosensor. Based on the aforementioned advantages, the electrochemical immunosensor proposed in this study has good repeatability, stability, high specificity, and a wide linear range, with a detection limit of 6.2 fg/ml. In the future, how to improve the preparation speed of the sensor and reduce the incubation time will become an important direction of the electrochemical immunosensor for practical detection.

## Data Availability

The original contributions presented in the study are included in the article/Supplementary Material, further inquiries can be directed to the corresponding author.
